# Smartphone usage during walking decreases the positive persistency in gait cycle variability

**DOI:** 10.1038/s41598-024-66727-1

**Published:** 2024-07-16

**Authors:** Shunpei Yano, Akihiro Nakamura, Yasuyuki Suzuki, Charles E. Smith, Taishin Nomura

**Affiliations:** 1 Department of Mechanical Science and Bioengineering, Osaka University, Osaka, 5608531 Japan; 2 Department of Statistics, North Carolina State University, Raleigh, NC 27695-8203 USA; 3 Department of Informatics, Kyoto University, Kyoto, 606-8501 Japan

**Keywords:** Central pattern generators, Biomedical engineering

## Abstract

Gait cycle variability during steady walking, described by the stride interval time series, has been used as a gait-stability-related measure. In particular, the positive persistency in the stride intervals with 1/*f*-like fluctuation and reduction of the persistency are the well-documented metrics that can characterize gait patterns of healthy young adults and elderly including patients with neurological diseases, respectively. Here, we examined effects of a dual task on gait cycle variability in healthy young adults, based on the mean and standard deviation statistics as well as the positive persistency of the stride intervals during steady walking on a treadmill. Specifically, three gait conditions were examined: control condition, non-cognitive task with holding a smartphone in front of the chest using their dominant hand and looking fixedly at a blank screen of the smartphone, and cognitive motor task with holding a smartphone as in the non-cognitive task and playing a puzzle game displayed on the smartphone by one-thumb operation. We showed that only the positive persistency, not the mean and standard deviation statistics, was affected by the cognitive and motor load of smartphone usage in the cognitive condition. More specifically, the positive persistency exhibited in the control and the non-cognitive conditions was significantly reduced in the cognitive condition. Our results suggest that the decrease in the positive persistency during the cognitive task, which might represent the deterioration of healthy gait pattern, is caused endogenously by the cognitive and motor load, not necessarily by the reduction of visual field as often hypothesized.

## Introduction

Falls are a public health issue in the aging and aged societies^1,2^. They could result in fatal and non-fatal injury and increase a risk of diminished quality of life after falls, particularly in older adults^3^. Moreover, falls of older adults impose a large economic burden in the U.S., which was about $50.0 billion as the annual medical cost attributable to fatal and nonfatal falls^4^, and probably also in other countries. Smartphone usage while walking, often referred to as the smartphone walking or the distracted walking, has become an extra dimension of a risk of falls for all generations, as smartphone ownership continues to climb^5^. Although smartphones made our lives dramatically convenient, it might also put us in danger. Kim et al. reported that people who are addicted to smartphones are more likely to have experienced falls compared with normal users^6^. Recent studies report that using a mobile phone to text while walking may compete with locomotor tasks, threat assessment and postural balance control mechanisms, which leads to an increased risk of accidental falls in young adults^7–9^. In this way, prevention of falls is a priority for our societies. Thus, development of measures that quantify gait stability for assessing a risk of falls is a pressing issue.

Gait cycle variability, i.e., fluctuation of the stride interval (stride time interval) from one stride to the next, is considered as one of the key indicators that contain crucial information about gait stability^10,11^. Older adults exhibit increased gait variability that is associated with fall history^12^, high-level gait disorders (HLGD)^13^ and neurodegenerative diseases^14,15^. However, very healthy older adults also exhibit increased gait variability^16^. Thus, it is difficult to quantify a risk of falls only by the magnitude (standard deviation or root mean square) of variability. Gait variability is not merely uncorrelated white noise but a time series with positive persistency with a 1/*f*-like power-law scaling, particularly in healthy young adults^17,18^. On the other hand, older adults exhibit less correlated, weaker positive persistency compared to young adults, which is associated with aging and neurodegenerative diseases^14,17–19^. In addition, HLGD patients with fall history exhibit less correlated variability than patients with no fall^13^ and the degree of reduction in the positive persistency is correlated with the severity of the disease^20,21^. Thus, the positive persistency can reflect the state of the neuromotor system as a nonlinear dynamical system, and it can be a biomarker to quantify the endogenous fall risk, gait stability and disease severity. However, we should note that the positive persistency in the stride intervals has been confirmed its predictive validity as a metric for gait stability merely in observational studies^22^.

Gait is not merely an automated motor activity that utilizes higher-level cognitive input^23–25^. A risk of falls increases among older adults with impaired cognitive function^26^ and patients with neurodegenerative diseases^27,28^, indicating cortical contributions to the control of upright posture^29^ and bipedal walking^30^, as revealed by activities and structure of the brain^31^. Moreover, older adults tend to encounter difficulties with performing a secondary cognitive task while walking, i.e., dual task walking^32^, which is often quantified by the magnitude of performance decline when conducting two tasks simultaneously^32,33^. In this way, difficulties in dual task walking in older adults can be characterized by larger performance declines, compared to young adults^34^. Declined performance in dual task walking have been related to increased fall risk^35^. Decline in performance and increase in fall risk might be caused when neural resources used for gait control become limited, i.e., when the neural resources are devoted to non-motor tasks^31^. The well-documented fact that dual tasks and attention demanding tasks during walking alter the gait variability, which indicates that the gait variability is one of the gait performances affected by higher-level cognitive functions in gait control. Previous studies using dual task walking suggested that cognitive load can affect gait performance even in healthy young adults^36–39^ as well as in older adults^32,33,40–42^. These studies investigated mean and magnitude of gait cycle variability, but there is little study that examined the effect of dual task on the positive persistency in the gait cycle variability. To be precise, there are several studies investigating the effect of cognitive load on the positive persistency in the gait variability^43,44^, but they have focused mainly on elderly people. That is, although it may seem that there are already many research reports examining the effect of dual task on the positive persistency in the gait cycle variability focused on healthy young adults, it is not the case. The current study aimed to provide firm evidence on this issue, using stride interval time series data with enough length for assessing the positive persistency, based on the scaling exponent of power-law distributed fluctuation, which can be achieved by the conventional detrended fluctuation analysis (DFA).

In the case of smartphone walking as one of the dual task walking paradigms, it is believed that the reduction of visual field and deterioration of awareness for surroundings lead to accidental falls^45^. In addition, it is expected that the cognitive load imposed by the smartphone usage could also lead to falls as described above. In this study, we investigated effects of smartphone usage while walking on the temporal dynamics of gait cycle variability, particularly on the positive persistency in the variability. Unlike previous studies that focused on the magnitude of gait variability^36,37,39^, this study focused on the temporal pattern of gait variability. In particular, we hypothesized that smartphone usage decreases the positive persistency in gait cycle variability. Specifically, we examined a possibility that it is the cognitive load by active use of a smartphone, but not just by the non-cognitive motor requirement to hold a smartphone in front of the body, that causes reduction of the visual field and deterioration of awareness for surroundings, decreases the positive persistency in gait cycle variability. Note that preliminary observations of this study have been reported elsewhere^46^. Based on the well-documented positive correlation between the decreased positive persistency and the fall risk in older adults and patients with neurodegenerative diseases, decrease in the positive persistency during smartphone walking would suggest that smartphone usage during walking decreases gait adaptability, and possibly increases a risk of falls.

## Methods

### Participants

Forty-four volunteers (22.6 ± 2.1 years, 39 males, 5 females) participated in this study. Participants signed an informed consent form approved by the ethical committee for human studies at Graduate School of Engineering Science, Osaka University. This study has been performed in accordance with the Declaration of Helsinki. All participants were free of disorders that impact gait.

### Apparatus and environment

Participants walked on an indoor treadmill (Bertec, Ohio, US) while wearing a safety harness to reduce a risk of injury due to falls. A Bluetooth-compatible 3-dimentionl accelerometer (TSND151, ATR-Promotions Co., Ltd., Kyoto, Japan) was taped on the right calcaneus. Range for measuring acceleration was set to ± 8G (G = 9.81 m/s^2^) and resolution was then 0.24 mG. The size of accelerometer is 40 mm × 50 mm × 14 mm and it weights 27 g. Raw acceleration data were recorded at 1000 Hz during walking.

### Procedure

**Figure 1:**
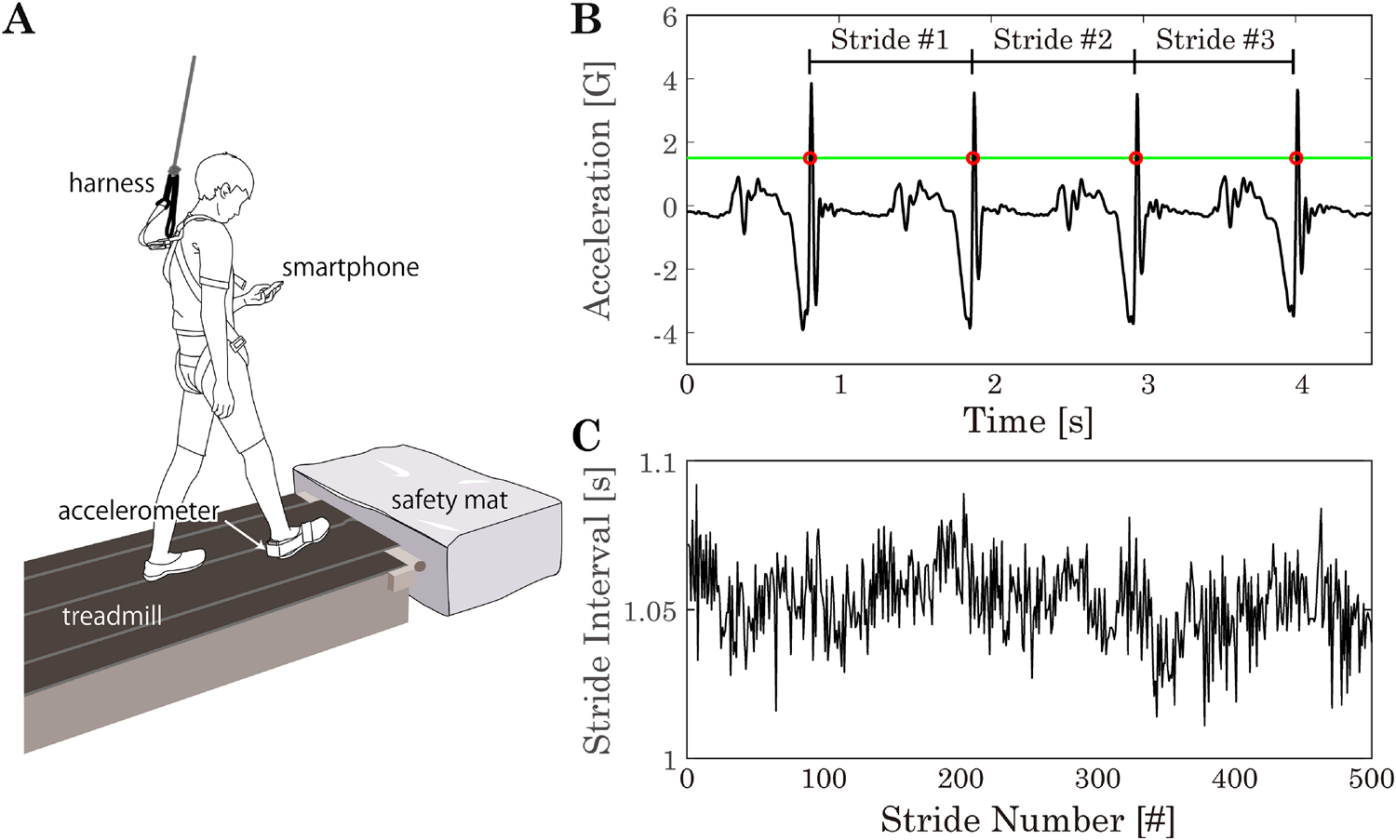
Acquiring stride intervals (SIs) from acceleration data. (**A**) Experimental procedure, including a human subject performing the cognitive task. (**B**) Example of accelerometer data in the direction of heel to toe for about 4 s. The heel to toe direction in the local coordinate of accelerometer is shown as upward direction in this graph. The green line and red circles are the acceleration threshold and the identified heel-strike timings, respectively. Heel-strike timings were defined as the data points which crossed the acceleration threshold value in the upward direction. The stride intervals (SIs), corresponding to the time between any two adjacent heel-strike timings, were computed to obtain SI time series. (**C**) Example of SI time series data for 500 strides during control task.

The experiment consisted of three gait conditions: control, non-cognitive and cognitive conditions. The self-selected preferred walking speed was used commonly across all three conditions for each participant. To this end, participants went through a preliminary run, going through each of three gait conditions for 1–2 min prior to the experiment, and decided on the preferred walking speed by which they could walk most comfortably. Because of the way of selecting walking speed, the walking speed adopted for three conditions tended to be too low for the healthy young individuals for the control condition. During the control condition, participants were instructed to walk steadily facing straight ahead. In the non-cognitive task condition, participants walked while holding a smartphone in front of their chest using their dominant hand and looking fixedly at a blank screen of the smartphone. This condition was set for the purpose of discriminating the effect of the non-cognitive motor requirement to hold the smartphone together with a reduction of the visual field from the effect of a cognitive load that was superposed on this condition. In the cognitive task condition, participants walked while holding a smartphone as in the non-cognitive task and playing a puzzle game 2048 (Solebon LLC) displayed on the smartphone by one-thumb operation. In each condition, participants walked for 30 min, with 20 min non-walking breaks between conditions. See Fig. 1A that illustrates the experimental procedure, including a human subject performing the cognitive task on the treadmill. The first 12 participants walked in the sequential order of control, non-cognitive task, and cognitive task conditions. This was because we expected that walking steadily on the treadmill for 30 min with the non-cognitive and cognitive tasks might not be easy, and performing easier tasks first and then performing the most difficult cognitive task at the end after adapting the non-cognitive task would be helpful for performing three tasks safely. However, we found that this was not the case, and participants could perform the cognitive task with no difficulty nor danger from the beginning. Thus, we instructed the next 12 participants to perform the tasks in the reverse order, namely in the order of cognitive task, non-cognitive task and control conditions to keep balance for the conditions. Then, the sequential order of conditions for the remaining 20 participants were allocated pseudo randomly. Specifically, each of the following sequential orders, control-cognitive-noncognitive, cognitive-control-noncognitive, noncognitive-cognitive-control, and noncognitive-control-cognitive, was used randomly with equal probability. Namely, 5 out of the 20 participants performed trials with 3 conditions using the one of those 4 sequential orders. Note that no instruction for prioritization of one of the tasks (walking vs other tasks) was given in non-cognitive and cognitive task conditions. In order to overcome the problematic issue caused by the substandard experimental design for the sequential order, we examined possible effects of the sequential orders statistically in the data analysis.

### Cognitive task

In the cognitive task condition, participants played the puzzle game 2048 as a cognitive task. 2048 is a game played on a gray 4-by-4 square grid with tiles numbered with powers of 2. Every turn, a player moves tiles up, down, left or right direction by swiping a screen of the smartphone. Tiles slide as far as possible in the chosen direction until they are stopped by either another tile or the edge of the grid. If tiles with an identical number collide, they will merge into a single tile numbered with their sum. Then, a tile of value 2 or 4 appears randomly at a vacant grid. The primary goal of the game is to create a tile that is numbered with the value 2048. The game ends when there are no vacant grids and no adjacent tiles with identical numbers. Players need to swipe the screen of the smartphone with their finger for moving tiles. In this study, participants were instructed to perform it by one-thumb operation. Therefore, this cognitive task is also an attention-demanding “motor” task. In this way, participants were instructed to play the game and walk simultaneously and to restart the game when the game ends during the measurement.

### Data analysis

Acceleration data were processed using MATLAB (Mathworks, Natick, MA). Statistical analyses were performed in R (The R Project for Statistical Computing).

### Extraction of stride intervals

We analyzed data of the acceleration in the direction of heel to toe in the local coordinate of the accelerometer. In order to minimize any start-up and end-up transient effects, we removed the first and last 30 s data from each time series data. The measured acceleration data were filtered with a 4th order low-pass Butterworth filter with a cut off frequency of 50 Hz. Figure 1B shows such a low-pass filtered acceleration time series. Positive and negative acceleration peaks, which correspond to the directions of toe to heel and heel to toe respectively, were observed before and after the heel strike timing. Therefore, we identified a data point that crossed an acceleration threshold value between two adjacent acceleration peaks as the heel-strike timing. The acceleration threshold values were determined visually for each acceleration time series. Time intervals between two successive identified heel-strike timings were calculated to obtain the stride interval (SI) time series. Figure 1C exemplifies such a SI time series.

### Stride interval correlations

To determine the degree of positive persistency in the SI time series, we applied a detrended fluctuation analysis (DFA) to each time series dataset. DFA is a modified random-walk analysis that utilizes the fact that a time series with long-range correlation become self-similar process by simple integration^25,47^. DFA has two advantages: it reduces noise effects and is insensitive to non-stationarities because the DFA process removes local trends^25^. To this end, we integrated a SI time series $x\left(k\right)$ with $k$ being the stride number, $k=1,\cdots ,N$, where $N$ is the total number of strides. Then, the integrated time series was separated into windows of length $n$, and then we computed a local trend function ${y}_{n,i}\left(k\right)$ in the *i*-th window to obtain ${y}_{n}\left(k\right)$ that connects sequentially the ordered local trend functions ${y}_{n,i}\left(k\right)$ across all windows ($i=1,\cdots ,N/n$). The average of the root mean square of $y\left(k\right)$ about local trend ${y}_{n}\left(k\right)$ was computed as

$$F\left(n\right)=\sqrt{\frac{1}{N}\sum_{k=1}^{N}{\left[y\left(k\right)-{y}_{n}\left(k\right)\right]}^{2}},$$

for a variety of window sizes $n$. The function $F\left(n\right)$ represents a relationship between the average fluctuation $F\left(n\right)$ as a function of window size $n$. Typically, $F\left(n\right)$ will increase with n. The fluctuations can be characterized by the scaling exponent $\alpha$, which is determined by the slope of the linear relationship between $\text{log}F\left(n\right)$ and $\text{log}n$. For the original process $x\left(k\right)$ in which the value at one time step has no correlation with any previous values, i.e., white noise, the integrated process is a classical Brownian motion, for which $\alpha$ = 0.5. In the case of $\alpha$ > 0.5, the process has a positive persistency, and if the relation between $\text{log}F\left(n\right)$ and $\text{log}n$ is truly linear, the process is said to be long-range correlated. In this study, we used a 2nd order polynomial fit (in the least squares sense) to evaluate the local trends and $F\left(n\right)$ for the window sizes spanning 6–500 cycles. The scaling exponent was computed by a linear regression line evaluated for the scaling regions spanning 30–200 cycles.

### Statistical tests

A multivariate repeated measures analysis with one within-subject factor (conditions) and one between-subject factor (orders) was conducted to evaluate the effects of the two factors and their interaction on our three dependent variables, mean of the SI referred to as mean SI, standard deviation of the SI referred to as SD of SI, scaling exponent α of the SI, using a tool developed recently^41^. Upon finding significant multivariate effects, one-way repeated measures ANOVAs were conducted for each dependent variable to identify which specific variables contributed to these effects. For significant ANOVA results, post-hoc pairwise comparisons using Bonferroni corrected paired *t*-tests were performed. A *p* value less than 0.05 was considered to be statistically significant in these tests.

## Results

**Figure 2:**
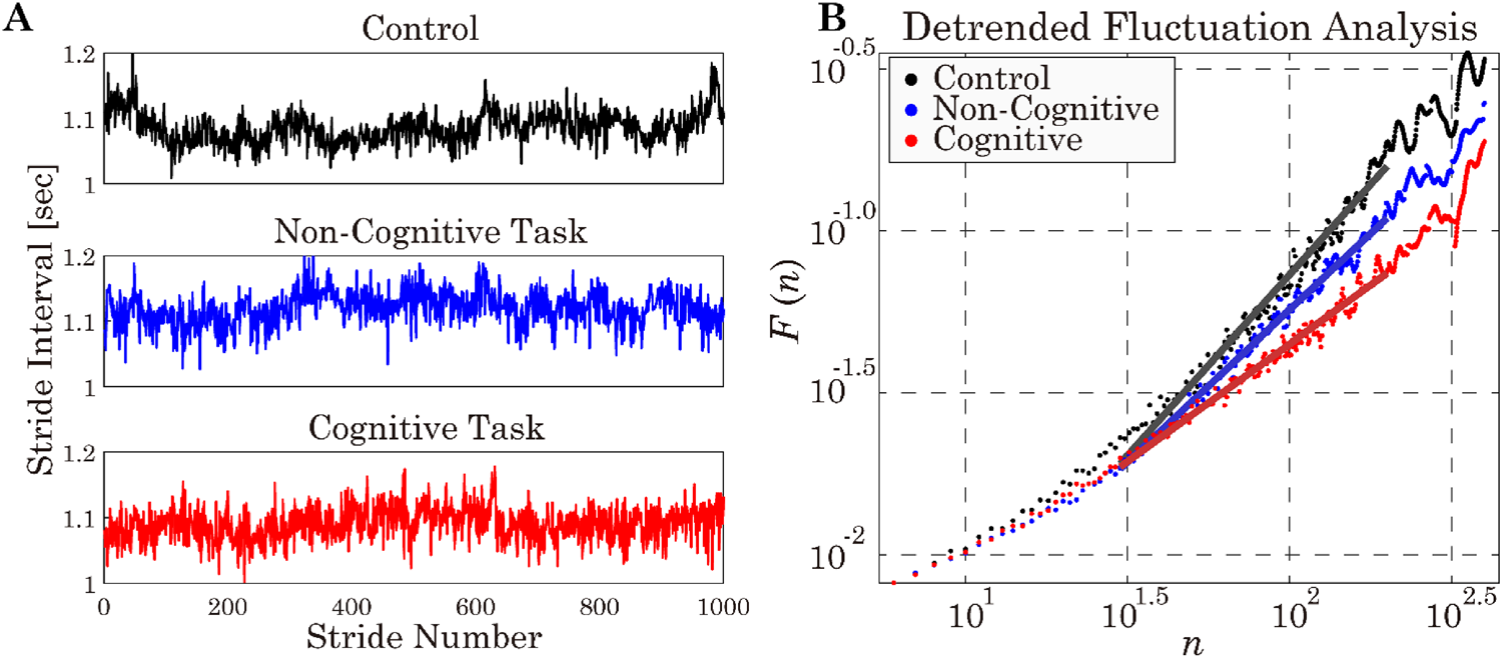
Examples of stride interval time series (**A**) and DFA plots (**B**) for a representative participant during each of three walking conditions. Solid lines in (**B**) represent the linear regression line for the plots of $\text{log}F\left(n\right)$ vs $\text{log}n$ for the scaling region. DFA shows that the stride interval fluctuations $F\left(n\right)$ increase with the shallower slope with time scale n increases in the cognitive task condition, compared to the other conditions. For this participant, the scaling exponent $\alpha$ was 0.72 for the cognitive task, whereas it was 0.94 for the non-cognitive task and 1.11 for the control.

The selected preferred walking speeds were between 0.8 and 1.2 m/s. One participant was derailed from the treadmill-belt shortly after the trial began in the cognitive task. However, because the participant could restart walking soon, we used data after the restart for the analysis. Figure 2A exemplifies the stride interval time series during each of the three conditions for a representative participant. While there were no markedly differences in the waveforms, it seems that the SI variability during the cognitive task might include less slow oscillatory components compared to the other two conditions (Fig. 2A). Indeed, the plots for DFA for this participant exhibited a shallower slope for the cognitive task, compared to the slopes that were close to unity for the other conditions (Fig. 2B). The observations shown in Fig. 2 were commonly shared by the other participants.

The multivariate method, two-way repeated measures MANOVA showed significant multivariate effects for the variable (conditions) with levels of control, non-cognitive, and cognitive (modified ANOVA-type statistic, MATS = 7.958, *p* = 0.016), indicating substantial overall impacts of these conditions on the dependent variables: mean SI, standard deviation (SD) of SI and scaling exponent α. Our findings indicate that the variable (orders) denoting the order of conditions had a minimal impact on the outcomes. Notably, the test statistics related to the order effects (MATS = 49.347, *p* = 0.376) and also the interaction between the variable conditions and variable orders (MATS = 13.999, *p* = 0.429) were not significant, suggesting that the sequence in which conditions were experienced by participants did not meaningfully alter their responses across the measured variables. Note that MATS denotes modified ANOVA Type Statistic^48^. The dependent variables, mean SI and SD of SI, were the only ones found to be significantly correlated (*r* = 0.36, *p* < 0.001).

**Figure 3:**
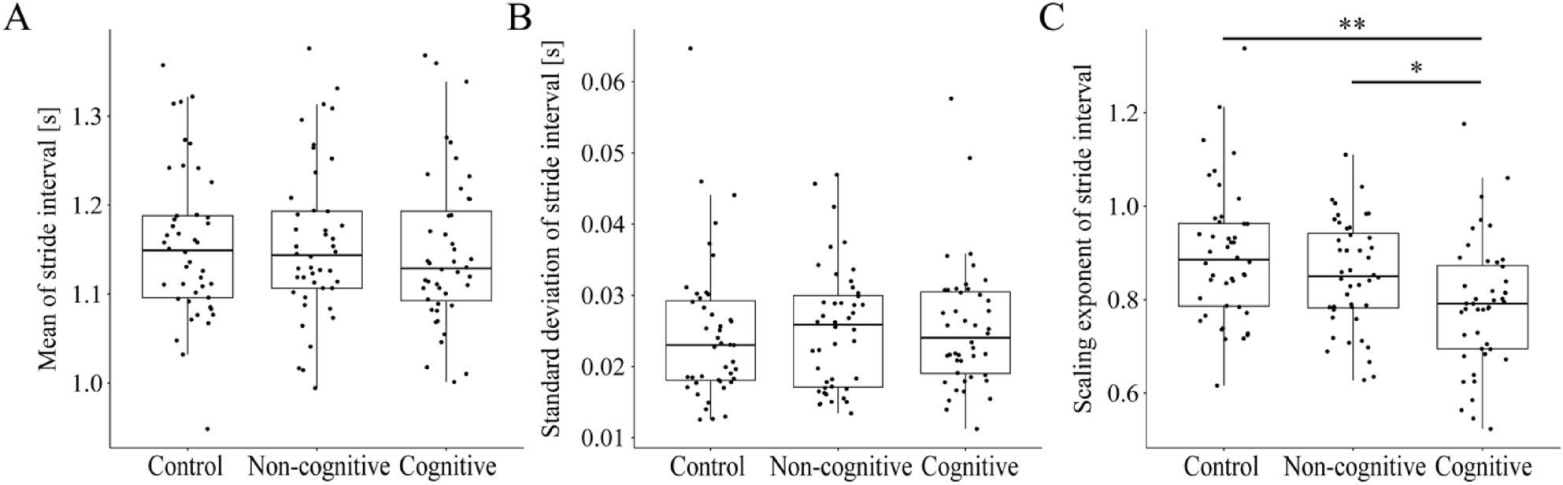
Box plots for the mean (**A**), the standard deviation (**B**) and the scaling exponent α of DFA (**C**) of stride intervals for each condition. One-way repeated measures ANOVAs for each dependent variable revealed significant effects of the conditions variable. Asterisks indicate statistically significant differences between conditions (*$p\le 0.05$, ** $p\le 0.01$). See text for further details.

Subsequent one-way repeated measures ANOVAs for each dependent variable revealed significant effects of the conditions variable. Specifically, for the scaling exponent α, there were significant differences observed across the control, non-cognitive, and cognitive conditions (F(2) = 9.704, *p* = 0.0002) as summarized in Table 1 and Fig. 3. As a result of the one-way repeated measures ANOVA, the main effect of the conditions variable was not significant for the response variable of mean SI (*F*(2) = 2.071, *p* = 0.132) and for the response variable of SD of SI (F(2) = 0.176, *p* = 0.839) as shown in Fig. 3A,B. Bonferroni corrected paired *t*-tests indicated significant differences between control and cognitive conditions (t(43) = 3.89, *p* < 0.001), and non-cognitive and cognitive (t(43) = 2.66, *p* < 0.005) for the scaling exponent α as shown in Fig. 3C. No significant differences were found between control and non-cognitive conditions (t(43) = 2.01, *p* = 0.153).

**Table 1:** One-way repeated measures ANOVAs outcomes.

Variables	df	F	*p*-value
Mean SI	2	2.071	0.132
Standard deviation of SI	2	0.176	0.839
Scaling exponent α	2	9.704	0.0002

## Discussion

We examined the effects of a dual task on gait cycle variability that was characterized by the mean SI and SD of SI statistics as well as the positive persistency (scaling exponent α) of the stride interval (SI) time series during walking on a treadmill. Specifically, three gait conditions were examined: control condition, non-cognitive task with holding a smartphone in front of the chest using their dominant hand and looking fixedly at a blank screen of the smartphone, and cognitive task with holding a smartphone as in the non-cognitive task and playing a puzzle game 2048 displayed on the smartphone by one-thumb operation. We showed that only the positive persistency quantified by the DFA scaling exponent α, but not the mean SI and SD of SI statistics, was affected by the cognitive and motor load of smartphone usage (Fig. 3, Table 1). More specifically, the positive persistency exhibited in the control and the non-cognitive conditions was significantly reduced in the cognitive condition. Some healthy participants in our study (seven out of 44) exhibited the scaling exponent slightly greater than 1.0. The most probable cause of the large exponent might be associated with the range (interval) used for the linear regression in DFA, where we used 30–200 cycles in this study. A use of larger upper cycle number for the DFA regression should result in a smaller exponent, since the positive persistency should be lost for any bounded process for a certain large time scale.

In this study, the mean SI and the SD of SI of the SI time series were not affected neither by the reduction of visual field nor by the cognitive load (Fig. 3A,B and Table 1). Our results are consistent with previous studies showing that healthy young adults exhibited no significant difference in the SD of SI by cognitive load^30,49^. On the other hand, other studies reported an increase in the SI during walking with a cognitive task^38^. Based on the assumption that attentional resources are limited^50^, changes in gait caused by dual tasks occur when cognitive resources necessary to perform two tasks simultaneously exceed a total capacity^51,52^. Beurskens et al. suggested that easy cognitive tasks, such that cognitive resource needed to perform the two tasks does not exceed total capacity, do not cause change in gait performance^34^. Playing 2048 used in the study might not be challenging enough to reach such a central capacity limit, because this task requires players only to swipe a screen and move a tile in horizontal and vertical directions. Therefore, our results might not contradict the previous studies that report the cognitive-load-induced increase in the SD of SI.

Playing 2048 is also an attention demanding motor task. Several studies reported that the SI were not affected by the motor task such as pressing electric buttons as many times as possible^53^ and transferring coins from the pocket attached on the right or left side of the hip to the pocket on the opposite side^54^. On the other hand, some studies reported effects of a motor task on the mean SI while walking with buttoning up four shirt buttons with a preferred hand^55^. As mentioned above for the cognitive load with and without significant effects, it was shown that the reduction of the SD of SI was correlated with better scores of a “catch game”, which is a attention demanding motor task, but the correlation did not exist for finger tapping, which is not a attention demanding motor task^56^. In this regard, playing 2048 might be a relatively simple and less attention-demanding motor task, because it needs only to swipe a screen of the smartphone and participants do not need to consider where to tap and how to control distance and direction of the swipe, leading to the absence of the changes in the mean SI and SD of SI statistics in our study.

The temporal structure of SI variability characterized by the scaling exponent α was affected by the usage of a smartphone in the cognitive task, but not by the reduction of visual field in the non-cognitive task. There were several studies that examined the effect of dual-tasking on temporal and spatial structure of gait variability^57^. Some studies reported that no significant difference was induced by a dual tasking in both the mean SI and SD of SI statistics as well as the scaling exponent α during walking^50^. Other studies reported that healthy and cognitively impaired older adults exhibited increased SD of SI by dual tasking of letter fluency task, where only healthy adults showed a reduction of the scaling exponent α^43^. It is interesting that the cognitive and attention-demanding motor task in this study affected the scaling exponent α, but not the mean SI and SD of SI statistics. As discussed above, the cognitive and motor load might not be challenging enough to affect the mean SI and SD of SI statistics, and compared to the mean SI and SD of SI, our results suggest that the positive persistency characterized by the scaling exponent α is a more sensitive biomarker to quantify available central capacity.

Falls during walking caused by usages of a smartphone are a healthcare issue^9^. However, possible causes of such falls have yet to be understood well. Our results suggest that the gait instability during the cognitive task was induced endogenously by the cognitive and motor load, not necessarily exogenously by the reduction of visual field as often hypothesized. A number of previous studies show that the positive persistency in the SI variability is correlated with endogenous fall history^14^ and the severities of neurological disease^21^, suggesting that it can be a biomarker to quantify the fall risk and gait stability^15,24^. Indeed, a previous study showed a correlation between the reduction of local dynamic stability, which was evaluated by Lyapunov exponent, and the reduction of the scaling exponent α^58,59^. Therefore, the positive persistency characterized by the scaling exponent α could be a good index that reflects endogenous gait stability/instability and a risk of falls. Because exogenous factors, such as stepped surface and surrounding pedestrians, could cause a fall easily, increase in the fall incident during walking with usages of a smartphone^9^ might be due to both endogenous and exogenous factors. However, note that the mechanisms of how the positive persistency is generated through a control process for stabilizing gait has yet to be clarified^60–62^, and thus also mechanisms of how the positive persistency is declined that lead to an increase in a risk of falls has not yet been established^61^.

This study has several limitations. The first limitation is about the experimental setup. That is, a thorough depiction of the secondary task outcomes was essential. The cognitive task performance and/or hand movement alongside gait should have been monitored to accurately assess dual-task performance. Because of the lack of the monitoring, quantitative characterization of the dual-task performance in this study created ambiguity. That is, the motor task in the non-cognitive task and that in the cognitive task may not be equivalent. Moreover, scores of the smartphone game, which were difficult to be recorded for repeated trials during every gait task, might have affected the level of cognitive task. The second limitation is a generalization of the current study to the real-world problem of smartphone walking. To keep walking on the treadmill, participants were imposed to walk at the constant speed whereas they keep and/or change gait speed actively during walking in the natural environment. Additionally, although we set a comfortable belt speed for each participant for all walking conditions, participants could walk at different speeds in a walking condition dependent manner in the real-world situation. Moreover, kinetics^63^ and kinematics^64^ of treadmill walking are slightly different from over-ground walking. Therefore, experiments on ground with more natural environment may lead to different outcomes.

Another limitation is a possible derailment from the treadmill. In this study, one participant derailed from the treadmill in the lateral direction during the cognitive task. Although the belt of the treadmill is sufficiently wide for performing steady gait, it might not be wide enough for smartphone walking for some people. The narrow path width seems to be a cause of relatively large variability of stride width detected by naked-eye observations. This could be another cause for the exponent α greater than unity for some cases in this study. Thus, examination of fluctuation in the heel strike positions would be an interesting future issue.

Further studies are still needed to understand mechanisms of how the positive persistency in health is generated and how it is reduced by diseases as well as by cognitive load. Measuring and accessing electroencephalogram-based brain activity during dual-tasking is one of the possible approaches for obtaining better understanding^25^. Moreover, several modeling studies have suggested that neural mechanisms of phase resetting control that modulate gait cycle play a key role for the emergence of the positive persistency^61,62^. Thus, examining a correlation between the phase resetting capability and the positive persistency is also an important issue to be addressed in the future study.

## Data Availability

The data that support the findings of this study are available from the corresponding author upon reasonable request.

## References

[1] Grisso JA, Schwarz DF, Wishner AR, Weene B, Holmes JH, Sutton RL (1990). Injuries in an elderly inner-city population. J. Am. Geriatr. Soc..

[2] Robinovitch SN, Feldman F, Yang Y, Schonnop R, Leung PM, Sarraf T, Sims-Gould J, Loughi M (2013). Video capture of the circumstances of falls in elderly people residing in long-term care: An observational study. Lancet.

[3] Sterling DA, O'Connor JA, Bonadies J (2001). Geriatric falls: Injury severity is high and disproportionate to mechanism. J. Trauma Acute Care Surg..

[4] Florence CS, Bergen G, Atherly A, Burns E, Stevens J, Drake C (2018). Medical costs of fatal and nonfatal falls in older adults. J. Am. Geriatr. Soc..

[5] Poushter J (2016). Smartphone ownership and internet usage continues to climb in emerging economies. Pew Res. Center.

[6] Kim H, Min J, Kim H, Min K (2017). Accident risk associated with smartphone addiction: A study on University Students in Korea. J. Behav. Addict..

[7] Pelicioni PHS, Chan LLY, Shi S, Wong K, Kark L, Okubo Y, Brodie MA (2023). Impact of mobile phone use on accidental falls risk in young adult pedestrians. Heliyon.

[8] Lin MIB, Huang Y-P (2017). The impact of walking while using a smartphone on pedestrians’ awareness of roadside events. Accid. Anal. Prev..

[9] Lee Y, Shin S (2024). Risk of using smartphones while walking for digital natives in realistic environments: Effects of cognitive–motor interference. Heliyon.

[10] Latash M (2000). There is no motor redundancy in human movements. There is motor abundance. Motor Control.

[11] Faisal AA, Selen LPJ, Wolpert DM (2008). Noise in the nervous system. Nat. Rev. Neurosci..

[12] Toebes MJP, Hoozemans MJM, Furrer R, Dekker J, Van Dieën JH (2012). Local dynamic stability and variability of gait are associated with fall history in elderly subjects. Gait Posture.

[13] Herman T, Giladi N, Gurevich T, Hausdorff JM (2005). Gait instability and fractal dynamics of older adults with a “cautious” gait: Why do certain older adults walk fearfully?. Gait Posture.

[14] Hausdorff JM, Mitchell SL, Firtion R, Peng CK, Cudkowicz ME, Wei JY, Goldberger AL (1997). Altered fractal dynamics of gait: Reduced stride-interval correlations with aging and Huntington’s disease. J. Appl. Physiol..

[15] Wu Y, Krishnan S (2010). Statistical analysis of gait rhythm in patients with Parkinson’s disease. IEEE Trans. Neural Syst. Rehabil. Eng..

[16] Kang HG, Dingwell JB (2008). Separating the effects of age and walking speed on gait variability. Gait Posture.

[17] Hausdorff JM, Peng CK, Ladin Z, Wei JY, Goldberger AL (1995). Is walking a random walk? Evidence for long-range correlations in stride interval of human gait. J. Appl. Physiol..

[18] Hausdorff JM, Purdon PL, Peng CK, Ladin Z, Wei JY, Goldberger AL (1996). Fractal dynamics of human gait: Stability of long-range correlations in stride interval fluctuations. J. Appl. Physiol..

[19] Hausdorff JM, Lertratanakul A, Cudkowicz ME, Peterson AL, Kaliton D, Goldberger AL (2000). Dynamic markers of altered gait rhythm in amyotrophic lateral sclerosis. J. Appl. Physiol..

[20] Warlop T, Detrembleur C, Bollens B, Stoquart G, Crevecoeur F, Jeanjean A, Lejeune T (2016). Temporal organization of stride duration variability as a marker of gait instability in Parkinson’s disease. J. Rehabil. Med..

[21] Ota, L., Uchitomi, H. Suzuki, K., Hove, M.J., Orimo, S. & Miyake, Y. Relationship between fractal property of gait cycle and severity of Parkinson’s disease. In *2011 IEEE/SICE International Symposium on System Integration (SII)*, 236–39 (2011). 10.1109/SII.2011.6147452.

[22] Bruijn SM, Meijer OG, Beek PJ, van Dieen JH (2013). Assessing the stability of human locomotion: A review of current measures. J. R. Soc. Interface.

[23] Yogev-Seligmann G, Hausdorff JM, Giladi N (2008). The role of executive function and attention in gait. Mov. Disord..

[24] Hausdorff JM (2009). Gait dynamics in Parkinson’s disease: Common and distinct behavior among stride length, gait variability, and fractal-like scaling. Chaos.

[25] Nakamura, A., Suzuki, Y., Yano, S. & Nomura, T. EEG activity related to decrease in persistency of gait cycle variability during distracted walking. In *2021 IEEE 3rd Global Conference on Life Sciences and Technologies (LifeTech)*, 183–184 (2021). 10.1109/LifeTech52111.2021.9391888.

[26] American Geriatrics Society, British Geriatrics Society, American Academy of Orthopaedic Surgeons Panel on falls prevention. Guideline for the prevention of falls in older persons interventions for preventing falls in the elderly. *J. Am. Geriatr. Soc.*, **49**, 664–72 (2001). 10.1046/j.1532-5415.2001.49115.x. 11380764

[27] Bloem BR, Hausdorff JM, Visser JE, Giladi N (2004). Falls and freezing of gait in Parkinson’s disease: A review of two interconnected, episodic phenomena. Mov. Disord..

[28] Pickering RM, Grimbergen YAM, Rigney U, Ashburn A, Mazibrada G, Wood B, Gray P, Kerr G, Bloem BR (2007). A meta-analysis of six prospective studies of falling in Parkinson’s disease. Mov. Disord..

[29] Nakamura A, Miura R, Suzuki Y, Morasso P, Nomura T (2023). Discrete cortical control during quiet stance revealed by desynchronization and rebound of beta oscillations. Neurosci. Lett..

[30] Koenraadt KL, Roelofsen EG, Duysens J, Keijsers NL (2014). Cortical control of normal gait and precision stepping: an fNIRS study. Neuroimage.

[31] Hupfeld KE, Geraghty JM, McGregor HR, Hass CJ, Pasternak O, Seidler RD (2022). Differential relationships between brain structure and dual task walking in young and older adults. Front. Aging Neurosci..

[32] Smith E, Cusack T, Blake C (2016). The effect of a dual task on gait speed in community dwelling older adults: A systematic review and meta-analysis. Gait Posture.

[33] Bayot M, Dujardin K, Dissaux L, Tard C, Defebvre L, Bonnet CT, Allart E, Allali G, Delval A (2020). Can dual-task paradigms predict falls better than single task? A systematic literature review. Neurophysiol. Clin..

[34] Beurskens R, Steinberg F, Antoniewicz F, Wolff W, Granacher U (2016). Neural correlates of dual-task walking: Effects of cognitive versus motor interference in young adults. Neural Plast..

[35] Montero-Odasso M, Verghese J, Beauchet O, Hausdorff JM (2012). Gait and cognition: A complementary approach to understanding brain function and the risk of falling. J. Am. Geriatr. Soc..

[36] Ebersbach G, Dimitrijevic MR, Poewe W (1995). Influence of concurrent tasks on gait: A dual-task approach. Percept. Motor Skills.

[37] Weerdesteyn V, Schillings AM, Duysens J, Van Galen GP (2003). Distraction affects the performance of obstacle avoidance during walking. J. Motor Behav..

[38] Beauchet O, Dubost V, Herrmann FR, Kressig RW (2005). Stride-to-stride variability while backward counting among healthy young adults. J. NeuroEng. Rehabil..

[39] Grabiner MD, Troy KL (2005). Attention demanding tasks during treadmill walking reduce step width variability in young adults. J. NeuroEng. Rehabil..

[40] Lindenberger U, Marsiske M, Baltes PB (2000). Memorizing while walking: Increase in dual-task costs from young adulthood to old age. Psychol. Aging.

[41] Li KZH, Lindenberger U, Freund AM, Baltes PB (2001). Walking while memorizing: Age-related differences in compensatory behavior. Psychol. Sci..

[42] van Iersel MB, Ribbers H, Munneke M, Borm GF, Rikkert MGO (2007). The effect of cognitive dual tasks on balance during walking in physically fit elderly people. Arch. Phys. Med. Rehabil..

[43] Lamoth CJ, van Deudekom FJ, van Campen JP, Appels BA, de Vries OJ, Pijnappels M (2011). Gait stability and variability measures show effects of impaired cognition and dual tasking in frail people. J. NeuroEng. Rehabil..

[44] Sejdić E, Findlay B, Merey C, Chau T (2013). The effects of listening to music or viewing television on human gait. Comput. Biol. Med..

[45] Lin MB, Huang Y (2017). The impact of walking while using a smartphone on pedestrians’ awareness of roadside events. Accid. Anal. Prev..

[46] Yano, S., Dimalanta, L., Suzuki, Y. & Nomura, T. Fluctuation of stride time intervals during walking with smartphone. In *2019 IEEE 1st Global Conference on Life Sciences and Technologies (LifeTech 2019)*, 296–297 (2019). 10.1109/LifeTech.2019.8884072.

[47] Peng CK, Buldyrev SV, Havlin S, Simons M, Stanley HE, Goldberger AL (1994). Mosaic organization of DNA nucleotides. Phys. Rev. E.

[48] Friedrich S, Pauly M (2018). MATS: Inference for potentially singular and heteroscedastic MANOVA. J. Multivar. Anal..

[49] Grubaugh J, Rhea CK (2014). Gait performance is not influenced by working memory when walking at a self-selected pace. Exp. Brain Res..

[50] Craik FIM, Bialystok E (2006). Cognition through the lifespan: Mechanisms of change. Trends Cogn. Sci..

[51] Daniel K (1973). Attention and Effort.

[52] Woollacott M, Shumway-Cook A (2002). Attention and the control of posture and gait: A review of an emerging area of research. Gait Posture.

[53] Galletly R, Brauer S (2005). Does the type of concurrent task affect preferred and cued gait in people with Parkinson’s disease?. Aust. J. Physiother..

[54] O’Shea S, Morris ME, Iansek R (2002). Dual task interference during gait in people with Parkinson disease: Effects of motor versus cognitive secondary tasks. Phys. Ther..

[55] Yang Y-R, Chen Y-C, Lee C-S, Cheng S-J, Wang R-Y (2007). Dual-task-related gait changes in individuals with stroke. Gait Posture.

[56] Hausdorff JM, Yogev G, Springer S, Simon ES, Giladi N (2005). Walking is more like catching than tapping: Gait in the elderly as a complex cognitive task. Exp. Brain Res..

[57] Ahmadi S, Sepehri N, Wu C, Szturm T (2019). Comparison of selected measures of gait stability derived from center of pressure displacement signal during single and dual-task treadmill walking. Med. Eng. Phys..

[58] Terrier P, Dériaz O (2011). Kinematic variability, fractal dynamics and local dynamic stability of treadmill walking. J. NeuroEng. Rehabil..

[59] Terrier P, Dériaz O (2013). Non-linear dynamics of human locomotion: Effects of rhythmic auditory cueing on local dynamic stability. Front. Physiol..

[60] Dingwell JB, John J, Cusumano JP (2010). Do humans optimally exploit redundancy to control step variability in walking?. PLoS Comput. Biol..

[61] Fu C, Suzuki Y, Morasso P, Nomura T (2020). Phase resetting and intermittent control at the edge of stability in a simple biped model generates 1/f-like gait cycle variability. Biol. Cybern..

[62] Okamoto K, Obayashi I, Kokubu H, Senda K, Tsuchiya K, Aoi S (2022). Contribution of phase resetting to statistical persistence in stride intervals: A modeling study. Front. Neural Circuits.

[63] White SC, Yack HJ, Tucker CA, Lin H-Y (1998). Comparison of vertical ground reaction forces during overground and treadmill walking. Med. Sci. Sports Exerc..

[64] Riley PO, Paolini G, Della Croce U, Paylo KW, Kerrigan DC (2007). A kinematic and kinetic comparison of overground and treadmill walking in healthy subjects. Gait Posture.

